# Photo-Oxidative Stress in Plants: ROS Signaling, Damage Propagation, and Systems-Level Resilience

**DOI:** 10.3390/antiox15030371

**Published:** 2026-03-15

**Authors:** Xinguo Li, Sha Yang, Jialei Zhang, Shubo Wan

**Affiliations:** 1 Shandong International Cooperation Laboratory for Agricultural Germplasm Resource Innovation, Institute of Crop Germplasm Resources, Shandong Academy of Agricultural Sciences, Ji’nan 250100, China; xinguol@163.com (X.L.); yangsha0904@126.com (S.Y.); zhangjialei19@163.com (J.Z.); 2 Shandong Provincial Key Laboratory of Field Crop Physiology, Ecology, and Efficient Production (Under Construction), Ji’nan 250100, China

**Keywords:** photo-oxidative stress, reactive oxygen species, retrograde signaling, photoprotection, climate resilience

## Abstract

Photo-oxidative stress, resulting from an imbalance between light absorption and photosynthetic carbon utilization, poses a fundamental challenge to plant survival and productivity. This review synthesizes recent advances to present an integrated framework connecting reactive oxygen species (ROS) signaling, damage propagation, and systems-level resilience. We move beyond describing ROS as mere toxic byproducts to position them as central hubs in a complex, interconnected network. We integrate the specific sites of ROS generation, particularly ^1^O_2_ at PSII and H_2_O_2_ at PSI, with their distinct retrograde signaling pathways (e.g., EXECUTER, β-cyclocitral, and RES/RCS pathways) that reprogram nuclear gene expression. A systems perspective is then applied to reveal how initial photochemical damage propagates through a self-amplifying “vicious cycle” of impaired photosystem repair, lipid peroxidation, and protein oxidation, ultimately threatening cellular integrity. Counteracting this cycle is a multi-layered photoprotective arsenal including NPQ, alternative electron sinks (CEF, WWC), and an integrated antioxidant network, which we re-evaluate not as independent modules but as a coordinated, evolutionary-tuned defense system. We synthesize this knowledge to highlight a central paradigm for crop improvement: the pervasive growth–defense trade-off. Investment in photoprotection, while crucial for survival, diverts resources from yield, explaining why single-gene modifications often fail in the field. Therefore, we argue that future strategies must move beyond simply enhancing single components and instead focus on “optimizing the network”. We conclude by outlining how synthetic biology, multi-omics integration, and genomics-assisted breeding can be leveraged to fine-tune this integrated system, aiming to develop climate-resilient crops that balance productivity with survival in an increasingly volatile climate.

## 1. Introduction

Global food security is fundamentally dependent on the photosynthetic efficiency of major crop species. However, their productivity is increasingly threatened by climate change-induced abiotic stresses, which trigger photo-oxidative damage, a critical physiological bottleneck that constrains yield and resilience. Yet, it harbors a paradox, as harvesting light energy renders plants highly susceptible to damage from excess light [1,2,3,4,5,6,7]. When light absorption surpasses the capacity of carbon assimilation, the photosynthetic apparatus becomes over-excited, triggering reactive oxygen species (ROS) generation through photo-oxidation. Importantly, while solar irradiance may not increase per se, climate change elevates the frequency and intensity of conditions, such as drought, heatwaves, and the resulting stomatal closure, that create a perceived “excess” of light by uncoupling its capture from its utilization. Therefore, photo-oxidative stress serves as a predominant mechanistic link and a key physiological bottleneck through which these combined climate stressors impair photosynthesis and growth, significantly contributing to losses in agricultural yield and carbon sequestration under suboptimal conditions [8,9].

This paradigm has shifted dramatically, which is especially relevant for crop improvement. Research reveals ROS, particularly hydrogen peroxide (H_2_O_2_) and singlet oxygen (^1^O_2_), as integral components of sophisticated signaling networks [10,11]. Understanding ROS not as mere toxins but as integral signaling molecules opens new avenues for engineering climate resilience. The molecular mechanisms of photo-oxidation, therefore, represent prime targets for biotechnological and breeding interventions aimed at sustaining yield stability under volatile climates. This review synthesizes recent advances in the molecular mechanisms of photo-oxidation, with a focus on interpreting these fundamental discoveries through an applied lens. Our core objective is to move beyond a descriptive catalog of genes and pathways to present an integrated conceptual framework for understanding and engineering photo-oxidative stress resilience. We will address three key questions: (1) How do specific ROS signals, originating from distinct cellular sites, integrate with hormonal networks to orchestrate a tailored stress response? (2) How does initial photochemical damage propagate through an interconnected network of lipid peroxidation, protein oxidation, and repair failure, creating a self-amplifying “vicious cycle” that determines cell fate? (3) What are the functional synergies and critical evolutionary trade-offs within the plant’s multi-layered photoprotective arsenal, and how can understanding these trade-offs guide the development of next-generation, climate-resilient crop varieties? By framing the available evidence within this systems-level perspective, we aim to provide a roadmap for translating fundamental knowledge into strategies that sustain crop yield under the dynamic and combinatorial stress conditions of a changing climate.

## 2. ROS Generation, Spatiotemporal Signaling, and Retrograde Communication

**Figure 1:**
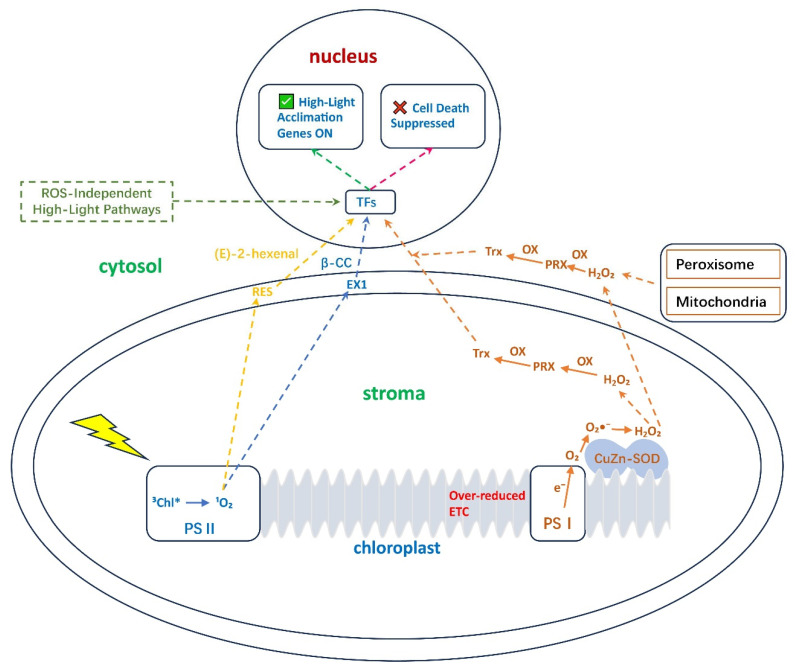
Integrated ROS Generation and Signaling Networks during Photo-oxidative Stress. Schematic of primary ROS generation sites in the chloroplast. At PSII, singlet oxygen (^1^O_2_) activates a specific signaling pathway via EXECUTER proteins and carotenoid derivatives like β-cyclocitral (β-CC), via Reactive electrophilic species (RES) like (E)-2-hexenal. At PSI, superoxide anions (O_2_•^−^) are dismutated to hydrogen peroxide (H_2_O_2_), which acts as a mobile signal, often relayed via the PRX/Trx redox network, to modulate nuclear gene expression. At other organelles, such as Peroxisome and Mitochondria, H_2_O_2_ is produced and via the PRX/Trx redox network to modulate nuclear gene expression. ROS-independent high-light pathways also influence gene expression. These distinct pathways allow the plant to tailor its response to the specific nature of the photo-oxidative stress. Abbreviations: EX1, EXECUTER1; PSI/II, Photosystem I/II; PRX, peroxiredoxin; Trx, thioredoxin; β-CC, β-cyclocitral; RES, Reactive electrophilic species.

The phenomenon of photo-oxidation is fundamentally rooted in the generation and management of ROS. Once considered merely toxic byproducts of aerobic metabolism, ROS are now recognized as pivotal signaling molecules that coordinate plant responses to environmental challenges. Understanding their precise sites of production, the specificity of their signals, and their role in systemic communication is essential to appreciate the complexity of photo-oxidative stress management (Figure 1).

### 2.1. Primary ROS and Their Specific Production Sites

ROS are inevitable by-products of electron transport in multiple cellular compartments, with chloroplasts as the primary source under photo-oxidative conditions. In the photosynthetic electron transport chain, Photosystem II (PSII) produces ^1^O_2_ via the interaction of O_2_ with the excited triplet state of chlorophyll (^3^Chl) when absorbed energy cannot be used for photochemistry [12,13]. ^1^O_2_ is highly reactive with a short half-life, confining its effects to the thylakoid membranes. Photosystem I (PSI) can generate superoxide anions (O_2_•^−^) primarily through the Mehler reaction when electron acceptors are limited. Under high light with strong photosynthetic control, over-reduction of the plastoquinone (PQ) pool can also become a significant source of O_2_•^−^ [14]. O_2_•^−^ is rapidly dismutated (spontaneously or by superoxide dismutase, SOD) to form H_2_O_2_, which is more stable and diffuses over longer distances, serving as a versatile signaling molecule [8,15,16,17].

Beyond chloroplasts, peroxisomes are a major site of H_2_O_2_ production in leaf mesophyll cells of terrestrial plants during photorespiration. The glycolate oxidase reaction in the canonical plant photorespiratory pathway is a key H_2_O_2_ generator under CO_2_-limiting conditions [18]. It is noteworthy that other photosynthetic organisms, such as many algae, utilize alternative photorespiratory routes that minimize peroxisomal H_2_O_2_ production, highlighting the diversity of metabolic adaptations to photo-oxidative challenges across lineages [19]. The glycolate oxidase reaction in photorespiration is a key H_2_O_2_ generator. The apoplast (cell wall space) also produces ROS via plasma membrane-localized NADPH oxidases (Respiratory Burst Oxidase Homologs, RBOHs), which are activated by stress to produce O_2_•^−^, later converted to H_2_O_2_ [20]. This apoplastic ROS wave is critical for systemic signaling, alerting distant tissues to localized stress.

### 2.2. Specificity of ROS Signals

A key advancement in plant biology is recognizing that different ROS, originating from distinct cellular sites and possessing unique chemical properties, trigger discrete genetic and metabolic programs [11,20]. This specificity allows plants to fine-tune their responses to the type and intensity of photo-oxidative stress. Unlike the limited direct effects of highly reactive ^1^O_2_ [13,21,22], H_2_O_2_ is more stable and mobile [15]. This fundamental spatial and chemical divergence establishes ^1^O_2_ and H_2_O_2_ as specific ligands that activate separate retrograde signaling cascades to reprogram nuclear gene expression [3], the detailed mechanisms of which are explored in the following section.

### 2.3. Retrograde Signaling

Retrograde signaling is the process that translates these specific ROS signatures from the chloroplast into adapted nuclear gene expression. This communication is essential for acclimation and involves dedicated pathways for each major ROS species.

The ^1^O_2_/EXECUTER Pathway: Due to its high reactivity and short diffusion distance, ^1^O_2_ does not reach the nucleus directly. Instead, it initiates a chloroplast-localized cascade mediated by the nuclear-encoded thylakoid proteins EXECUTER1 (EX1) and EXECUTER2 (EX2). Oxidation of EX1 by ^1^O_2_ triggers conformational changes or degradation, activating downstream signaling that involves chloroplast kinases and leads to the accumulation of secondary messengers such as β-cyclocitral (β-CC), a carotenoid oxidation derivative that translocates to modulate expression of nuclear stress-acclimation genes [12,23,24]. In parallel, ^1^O_2_ directly oxidizes polyunsaturated fatty acids in the thylakoid membranes, generating lipid peroxides. The breakdown of these peroxides produces a suite of reactive electrophile species (RES), such as (E)-2-hexenal and malondialdehyde. These small, diffusible reactive carbonyl species (RCS) act as critical retrograde signals, translocating to the nucleus where they can covalently modify transcription factors and histone proteins, directly altering the expression of defense and detoxification genes [25,26]. While EX1 is established as the primary sensor, the role of EX2 is becoming clearer. EX2 shares structural homology with EX1 and is also required for a full ^1^O_2_ response. However, recent work suggests its role may be more nuanced than simple redundancy. EX2 potentially modulates the threshold for ^1^O_2_ signaling or integrates specific environmental cues, such as temperature, to shape the acclimation response, ensuring it is appropriate for the exact stress combination encountered [27]. This integrated ^1^O_2_ signaling network, comprising the EX/β-CC pathway and the RES/RCS pathway, activates a specific genetic program, with outcomes ranging from photoacclimation to programmed cell death depending on signal flux. The EX1-mediated pathway orchestrates distinct nuclear responses; moderate ^1^O_2_ flux promotes acclimation genes, while sustained flux activates programmed cell death, acting as a metabolic reset mechanism [28,29,30].

The H_2_O_2_ Relay Pathways: In contrast, H_2_O_2_ functions as a mobile retrograde signal. It can diffuse from the chloroplast stroma into the cytosol, often via aquaporins, where it influences nuclear components [3]. The specificity of the H_2_O_2_ signal is achieved through multiple mechanisms: the oxidation of specific cysteine residues on target proteins (e.g., transcription factors like TGAs), its spatial origin, and critically, its intensity and duration [11,20]. This temporal dimension is emerging as a key integration point; transient H_2_O_2_ bursts trigger acclimation responses, while sustained elevation activates programmed cell death pathways [29]. The signal is relayed through redox relay networks involving peroxiredoxins (PRXs) and thioredoxins (Trxs), which transfer oxidative equivalents to transcriptional regulators [2,31,32]. These thiol peroxidases thus function not merely as scavengers but as signal receptors and transducers that convert quantitative ROS information into qualitative transcriptional outputs. Notably, EX2 has also been implicated in certain nuclear gene expression responses to H_2_O_2_, indicating it may function as a shared component that helps integrate signals from different ROS sources, adding a layer of complexity to the network’s regulation [20].

Integration with Classic Retrograde Signals: These ROS pathways interact with other retrograde signals linked to chloroplast metabolism. These include the redox state of the plastoquinone pool and stress-induced metabolites like 3′-phosphoadenosine 5′-phosphate (PAP) and methylerythritol cyclodiphosphate (MEcPP). For instance, MEcPP produced under high light activates specific stress-response genes and intersects with ROS signaling pathways, allowing the cell to fine-tune its acclimation strategy based on a comprehensive readout of chloroplast status [33].

### 2.4. Integration with Hormonal Signaling Networks

The intricate crosstalk between ROS and phytohormone pathways forms a sophisticated, integrated signaling network essential for tailoring plant stress responses. This system is highly dynamic, with the nature of the interaction varying significantly depending on the specific stress combination, allowing the plant to prioritize and fine-tune its acclimation strategies [20].

The integration of hormonal and ROS signaling is pivotal in managing the onset and consequences of photo-oxidative stress. A key interaction involves abscisic acid (ABA) and H_2_O_2_. Under drought conditions, ABA-induced stomatal closure is a primary defense to conserve water. However, this concurrently triggers a cascade that leads to photo-oxidative stress by limiting CO_2_ availability for carboxylation. The resulting imbalance promotes over-reduction of the photosynthetic electron transport chain (PETC), favoring H_2_O_2_ production at PSI. This chloroplast-derived H_2_O_2_, in turn, can amplify ABA signaling, creating a feed-forward loop that reinforces stomatal closure and coordinates the expression of shared stress-response genes [1,20]. Thus, the ABA-H_2_O_2_ module exemplifies how a hormonal response to one stress (drought) directly instigates the physiological conditions for photo-oxidative stress, while simultaneously orchestrating a coordinated acclimation response.

Biotic stress responses are orchestrated by more specific ROS-hormone pairings. Salicylic acid (SA)-mediated systemic acquired resistance (SAR) against biotrophic pathogens is closely linked to H_2_O_2_ signaling [34,35]. In contrast, ^1^O_2_ acts as a potent activator of jasmonic acid (JA)-mediated defenses, which are crucial against necrotrophs and herbivores. This connection is mechanistically grounded in the shared origin of JA and ^1^O_2_-derived signals from chloroplast lipids. ^1^O_2_ directly peroxidizes chloroplast membrane lipids, generating a pool of lipid hydroperoxides. These are precursors for both jasmonates (via the octadecanoid pathway) and various RES, such as phytoprostanes and specific reactive carbonyls (e.g., (E)-2-hexenal). These RES themselves can act as mobile stress signals that upregulate JA biosynthesis genes and modulate JA-responsive transcription factors [36,37]. Therefore, ^1^O_2_ does not merely coincide with JA activation; it initiates the synthesis of both JA and complementary RES signals from a common lipid peroxidation substrate, creating an integrated defensive output. This explains how ^1^O_2_ generated during abiotic photo-oxidative stress can pre-emptively prime JA-dependent defenses, anticipating potential opportunistic necrotrophic infection following cellular damage [1].

The integration of these pathways reveals a context-dependent hierarchy. Under combined stress, such as high light with pathogen attack, initial ^1^O_2_ from damaged PSII may prime JA signaling against necrotrophs. A subsequent biotrophic infection, however, could trigger a dominant SA response that antagonistically suppresses the JA pathway, illustrating the complex and sometimes conflicting priorities within the network [38]. Recent multi-omics studies, integrating transcriptomics, proteomics, and metabolomics, have begun to deconvolute the complexity of the ROS-hormone interactome, revealing regulatory hubs beyond the core ABA/SA/JA framework. For instance, brassinosteroids (BRs) have emerged as key modulators of ROS homeostasis. A time-resolved multi-omics analysis of high-light stress in *Arabidopsis* identified the transcription factor BES1, a key BR signaling component, as a central node. BES1 directly regulates a suite of genes encoding both ROS-producing (e.g., RBOHs) and ROS-scavenging (e.g., peroxidases) enzymes, positioning BR signaling as a dynamic rheostat for cellular redox balance [39]. Similarly, integrative network analysis has linked auxin signaling with H_2_O_2_-mediated root responses, identifying specific *SMALL AUXIN UP-RNA* (*SAUR*) genes that are rapidly induced by ROS and may modulate cell expansion under stress [40]. Furthermore, metabolomic profiling has highlighted the role of pipecolic acid, a systemic defense potentiator, in amplifying ROS signals during systemic acquired acclimation, suggesting that this metabolite acts as a mobile integrator of redox and hormonal cues across tissues [41]. These findings underscore that the ROS-hormone network is a highly interconnected web rather than a set of linear pathways. Future research leveraging single-cell omics and CRISPR-based perturbation screens will be crucial to define the spatial specificity and hierarchy of these newly identified hubs within the living plant under combinatorial stress.

This regulatory hierarchy ensures the plant’s limited resources are allocated toward the most immediate threat, demonstrating the precision and adaptability of the interconnected ROS-phytohormone communication system.

From above, synthesizing these diverse signaling pathways reveals a fundamental principle: the chloroplast acts as a computational hub that integrates multiple input signals, ROS identity (^1^O_2_ vs. H_2_O_2_), signal intensity (flux), duration (transient vs. sustained), and context (coincident hormonal signals), to generate a tailored output response. The EXECUTER pathway provides high-sensitivity ^1^O_2_ detection, the RES/RCS pathway amplifies and propagates lipid peroxidation signals, and the PRX/Trx network transduces H_2_O_2_ information while simultaneously monitoring the cell’s broader thiol redox state. This integrated signaling system allows plants to distinguish between mild stress requiring acclimation and severe stress demanding cell death, a decision point with profound implications for whole-plant fitness.

## 3. Mechanisms of Photo-Oxidative Damage

**Figure 2:**
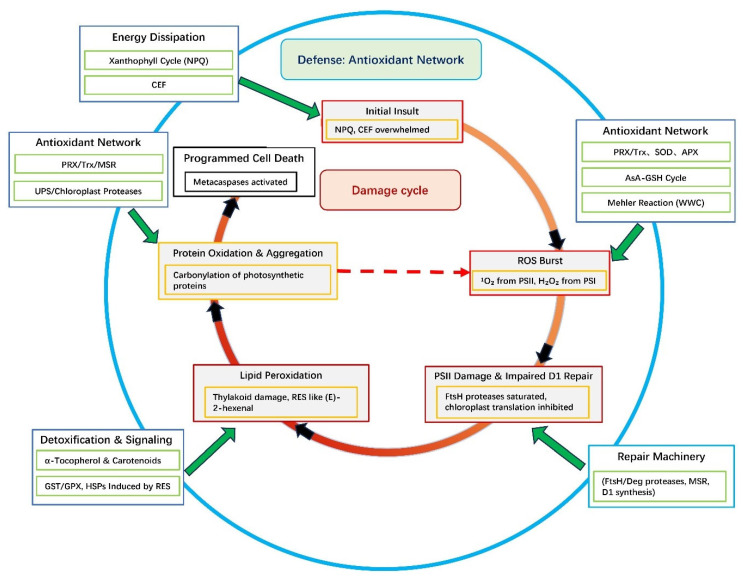
The vicious cycle of photo-oxidative damage and integrated defense interventions. A systems view of photo-oxidative damage showing how an initial failure of photoprotection triggers a self-amplifying vicious cycle (inner grey/black arrows). Key damage events are interconnected: (1) PSII repair impairment increases ^1^O_2_ production; (2) ^1^O_2_ and •OH cause lipid peroxidation, generating damaging RES; (3) ROS and RES cause protein oxidation and aggregation. Crucially, this protein damage impairs the cell’s repair and detoxification systems (e.g., proteasome, chaperones), which feeds back to exacerbate the initial photo-oxidative insult, closing the loop. Surrounding this cycle are the multi-layered defense systems (outer ring) that act at specific points to contain or break the cycle. Abbreviations: APX, ascorbate peroxidase; AsA, Ascorbate; CEF, cyclic electron flow; GSH, Glutathione; GST, glutathione s-transferase; GPX, glutathione peroxidase; HSP, heat shock protein; MSR, Methionine sulfoxide reductases; PSI/II, Photosystem I/II; PRX, perox-iredoxin; NPQ, non-photochemical quenching; RES, Reactive electrophilic species; SOD, superoxide dismutase; Trx, thioredoxin; WWC, water–water cycle; UPS, ubiquitin–proteasome system.

When photoprotective and antioxidant capacities are overwhelmed, ROS cause widespread cellular damage. Critically, this damage is not a collection of independent events but rather a self-propagating systems-level failure in which each destructive process amplifies the others, creating a vicious cycle (Figure 2). Understanding this cycle as an emergent property of interconnected networks, rather than as linear cause-and-effect, is essential for predicting when cells will transition from reversible damage to irreversible collapse.

### 3.1. An Interconnected Damage Network

As illustrated in Figure 2, the cycle is initiated at multiple possible entry points. However, the PSII repair cycle serves as a central node because its failure directly increases ^1^O_2_ production [12,42,43], which in turn drives both lipid peroxidation [25,44] and inhibition of new protein synthesis [45,46]. Lipid peroxidation products (e.g., RES) then exacerbate protein damage [25,26], while protein oxidation inactivates the very repair and detoxification systems (proteasome, chaperones, antioxidant enzymes) needed to break the cycle. This positive feedback architecture explains why damage accelerates once a threshold is crossed.

The initial site of photo-oxidative damage often depends on the stress type and the predominant ROS species. However, the ensuing effects are highly synergistic, creating a self-propagating cycle of deterioration. A central node in this network is the impairment of the PSII repair cycle.

When the D1 protein repair machinery (e.g., FtsH protease) becomes saturated or itself oxidized under sustained stress [12,42,43], the accumulation of non-functional PSII centers leads to a direct increase in ^1^O_2_ production [13]. This ^1^O_2_ burst has two major consequences that propagate the damage.

The first is direct exacerbation of lipid peroxidation. The thylakoid membranes, rich in polyunsaturated fatty acids (PUFAs), are prime targets for ^1^O_2_ and hydroxyl radicals. Increased lipid peroxidation compromises membrane integrity, further destabilizing the photosynthetic apparatus and potentially inactivating membrane-bound protein complexes [23,44].

The second is the inhibition of de novo protein synthesis. The oxidative by-products of lipid peroxidation, such as RES like (E)-2-hexenal, as well as H_2_O_2_ itself, can diffuse to the stromal compartment and inhibit chloroplast translation [45,46]. This creates a critical bottleneck, preventing the synthesis of new D1 and other proteins essential for repair, thereby locking the cycle in a destructive feedback loop.

Simultaneously, damage to PSI, which is often caused by acceptor-side limitation and over-reduction, can be irreversible due to the destruction of its Fe-S clusters [17,47]. Photoinhibition of PSI halts electron flow through this central complex, thereby crippling cyclic electron flow (CEF), a key photoprotective mechanism for regulating the proton motive force and preventing over-reduction [15,48]. The failure of CEF further exacerbates the over-reduced state of the electron transport chain, increasing ROS production at both photosystems and accelerating the damage to lipids and proteins [49].

This convergence of damage on the proteostasis network represents a final common pathway. The accumulation of carbonylated and aggregated proteins, despite the efforts of peroxiredoxins and the ubiquitin–proteasome system, ultimately leads to the activation of metacaspases and the initiation of programmed cell death [50]. This organized death removes cells that are net sinks of energy and potential sources of propagating oxidative signals, protecting the rest of the organism.

### 3.2. Lipid Peroxidation and Oxylipin Signaling

Beyond their role in structural damage, the peroxidation of thylakoid lipids constitutes a major source of retrograde signaling molecules. In particular, •OH and ^1^O_2_ initiate the peroxidation of PUFAs, forming lipid hydroperoxides. These unstable compounds decompose into a variety of RES, also known as RCS, such as malondialdehyde (MDA) and (E)-2-hexenal [25,37,44,51]. These small, diffusible RES act as key secondary messengers, complementing the EX/β-cc pathway to transduce the ^1^O_2_ signal from the chloroplast to the nucleus [25,26].

This degradation has dual effects. It compromises membrane structure and photosynthetic efficiency, and RES (oxylipins) acts as signaling molecules. Oxylipins like (E)-2-hexenal covalently modify proteins (protein carbonylation) and activate defense-related gene expression. For instance, (E)-2-hexenal directly induces the expression of genes encoding *GLUTATHIONE S-TRANSFERASE* (*GST*) and *HEAT SHOCK PROTEINS* (*HSPs*) [37,52,53]. GSTs are crucial for detoxifying electrophiles and ROS, while HSPs protect and refold damaged proteins. Furthermore, transcriptomic studies reveal that (E)-2-hexenal and related RES activate a specific suite of transcripts that overlaps with, but is distinct from, H_2_O_2_-induced genes, including those involved in the synthesis of protective flavonoids and glucosinolates [54]. This demonstrates that membrane lipid peroxidation is not merely a degenerative process but is transduced into a coordinated genetic defense program for acclimation.

### 3.3. Proteostasis and Protein Degradation

Maintaining proteome integrity (proteostasis) is critical under stress, as photo-oxidation causes irreversible protein carbonylation, leading to loss of function, aggregation, and cytotoxicity.

Plants defend against protein oxidation via the antioxidant system, with 2-Cys PRXs as key H_2_O_2_ and organic hydroperoxide scavengers in chloroplasts [31]. Under severe stress, 2-Cys PRXs become overoxidized, switching from peroxidases to molecular chaperones that prevent protein aggregation [32].

When prevention fails, damaged proteins are degraded via the ubiquitin–proteasome system (UPS) and proteases like FtsH. Methionine sulfoxide reductases (MSRs) specifically repair oxidized methionine residues in photosynthetic proteins [55], while metacaspases trigger controlled proteolysis or programmed cell death in extreme cases, removing heavily damaged cells to benefit the organism [50].

## 4. Anti-Oxidation and Photoprotective Mechanisms

Recent research has advanced understanding of these mechanisms, enabling predictive and engineering applications (Figure 2).

### 4.1. A Multi-Layered and Integrated Photoprotective Arsenal

To counteract photo-oxidative stress, plants employ an integrated, multi-tiered defense system that operates across temporal and functional scales. This coordinated arsenal includes the rapid dissipation of excess light energy, a sophisticated metabolic network for maintaining redox homeostasis, and the flexible redirection of photosynthetic electron flow. Together, these strategies function synergistically to prevent the over-reduction of PETC and mitigate cellular damage. Crucially, these three layers of defense are not deployed independently but are temporally and functionally coordinated. Non-photochemical quenching (NPQ) provides millisecond-to-second responses to sudden light fluctuations [56]. The AsA-GSH cycle and enzymatic antioxidants operate on seconds-to-minutes timescales, buffering against sustained imbalances [14,57]. Alternative electron sinks, particularly CEF and the water–water cycle (WWC), function continuously but become critical under prolonged stress when NPQ capacity is saturated [15,58,59]. This tiered response system ensures that mild, transient stresses are managed with minimal metabolic cost, while severe, sustained stresses trigger progressively more resource-intensive defenses, a strategy that optimizes the growth–defense trade-off discussed in Section 4.3.

#### 4.1.1. Dynamic Energy Dissipation

The first and fastest line of defense is dynamic energy dissipation through NPQ. In vascular plants, NPQ is activated by thylakoid lumen acidification under high light, which protonates the PSII Subunit S (PSBS) and stimulates the violaxanthin-to-zeaxanthin conversion [60]. This process induces conformational changes in antenna complexes, safely dissipating excitation energy as heat [61]. The kinetics of NPQ relaxation are critical; slow relaxation under fluctuating light can unnecessarily limit photosynthesis. Landmark research demonstrated that accelerating NPQ relaxation by overexpressing key components (xanthophyll cycle enzymes and PSBS) enhanced tobacco biomass yield by approximately 15%, validating the agricultural potential of optimizing this dynamic process [56]. The full induction of NPQ is further supported by the proton gradient sustained by cyclic electron flow, illustrating the crosstalk between regulatory pathways [15].

#### 4.1.2. Metabolic Redox Buffering

Underpinning rapid responses to ROS is a robust metabolic network centered on redox homeostasis. This network comprises both non-enzymatic antioxidants and a spatially organized suite of enzymatic components, each with distinct substrate specificities and subcellular localizations (summarized in Table 1). This compartmentalization ensures rapid detoxification at the site of ROS generation.

The ascorbate–glutathione (AsA-GSH) cycle forms the core of the chloroplast antioxidant system, functioning as a central redox hub. Within this cycle, H_2_O_2_ is scavenged by ascorbate peroxidase (APX), while ascorbate is regenerated via glutathione and NADPH. The localization of specific APX isoforms, such as thylakoid-bound tAPX, is crucial for local membrane protection [14,57]. This system is intrinsically linked to cellular metabolism through NADPH supply and is deeply integrated with the thioredoxin (Trx) system. Thioredoxins (Trx-f, -m, -x, -y, -z) regulate enzyme activity via dithiol-disulfide exchange, with Trx-x specifically activating peroxiredoxins. The Trx and GSH systems often operate synergistically, and their integration is mediated by key players like NADPH-thioredoxin reductase C (NTRC), which connects photosynthetic electron flow, antioxidant metabolism, and carbon assimilation [62].

SOD isoforms (Mn-SOD, Fe-SOD, Cu/Zn-SOD) provide the first line of enzymatic defense by dismutating O_2_•^−^ to H_2_O_2_ and O_2_ in chloroplasts, mitochondria, peroxisomes, and the cytosol. The resulting H_2_O_2_ is processed by different systems depending on its origin. In addition to the APX-dependent AsA-GSH cycle in chloroplasts, catalase (CAT) efficiently breaks down high fluxes of H_2_O_2_ produced during photorespiration in peroxisomes without consuming reductant. Glutathione peroxidases (GPXs) further contribute to H_2_O_2_ and organic hydroperoxide reduction, linking peroxide metabolism to the glutathione redox buffer. Peroxiredoxins (PRXs), particularly the chloroplast 2-Cys PRXs, serve as high-affinity peroxidases for H_2_O_2_ and peroxynitrite, and can switch to a chaperone function under severe oxidative load, preventing protein aggregation [31,32,63]. Finally, dedicated repair enzymes like methionine sulfoxide reductases (MSRs) reverse specific oxidative modifications to methionine residues in proteins, restoring function [55]. The synergistic and often redundant operation of this network is fundamental to maintaining redox homeostasis and preventing the cascade of damage described in Section 3.

#### 4.1.3. Alternative Electron Sinks

When light absorption exceeds carbon assimilation capacity, plants activate alternative electron sinks to alleviate PETC over-reduction. CEF plays a fundamental role by recycling electrons from ferredoxin back to the plastoquinone pool, primarily through the PGR5/PGRL1 and NDH-dependent pathways [15]. This process is essential for generating the trans-thylakoid proton gradient that drives ATP synthesis and activates NPQ, thereby providing crucial protection to PSI [15]. The WWC, which involves the Mehler reaction, serves as another electron sink by reducing oxygen to O_2_•^−^, which is subsequently detoxified to water via SOD and the AsA-GSH cycle, fulfilling roles in both electron dissipation and redox signaling [14,64]. Critically, the WWC functions as a vital electron valve that prevents the over-reduction of the PSI acceptor side. By providing an alternative electron pathway, it maintains a mild oxidizing environment around the Fe-S clusters, thereby directly preventing the conditions that lead to their destructive reduction. This role is paramount under fluctuating light, where sudden increases in irradiance can cause rapid over-reduction. The WWC, alongside CEF, acts as a rapid-response system to drain excess electrons, safeguarding PSI from irreversible photoinhibitory [58,59]. Additionally, photorespiration, once considered a wasteful pathway, is now recognized as an indispensable electron sink under CO_2_-limiting conditions. By consuming ATP and NADPH during the recycling of 2-phosphoglycolate, it effectively relieves over-reduction pressure on the PETC and is vital for plant survival under combined stress [65].

In summary, the plant’s photoprotective strategy is a highly integrated system where dynamic energy quenching, metabolic redox buffering, and flexible electron partitioning operate in concert. This multi-layered defense enables plants to balance efficient light utilization with the avoidance of photo-oxidative damage. The effectiveness of this integrated system, however, is shaped by evolutionary trade-offs between growth and defense, a concept explored in the following section.

### 4.2. The Repair Dimension for PSII and PSI

The multilayered preventative defenses described above, energy dissipation, redox buffering, and alternative electron sinks, are remarkably effective at preventing photodamage under most conditions. However, when stress intensity or duration overwhelms these systems, oxidative damage to the photosystems becomes inevitable. At this point, the plant must deploy its most resource-intensive defense strategy: the active repair and replacement of damaged photosynthetic components. The capacity for repair, particularly its contrast between the two photosystems, fundamentally determines whether a plant recovers from stress or succumbs to irreversible damage.

Sustained photo-oxidative stress targets both photosystems, but their capacity for repair and the consequences of failure are fundamentally different, dictating long-term resilience.

The repair of PSII is a high-frequency, dedicated cycle centered on the rapid turnover of the D1 protein. While this cycle is initiated by ^1^O_2_-mediated damage, its efficiency under prolonged stress is compromised at multiple points. The FtsH protease complex responsible for degrading damaged D1 can become saturated or inactivated by oxidation [42], while the concurrent inhibition of chloroplast translation by ROS and lipid peroxidation by-products (e.g., (E)-2-hexenal) starves the cycle of new D1 protein [46,66]. This leads to an accumulation of inactive PSII centers, escalating ROS production, and propelling the wider damage network described in Section 3.1.

In stark contrast, damage to PSI is often a terminal event. The over-reduction conditions prevalent under combined stresses (e.g., high light with low CO_2_) can destroy the Fe-S clusters (Fx, FA, FB) within the PSI reaction center. No efficient in situ repair mechanism exists for these complex cofactors; recovery requires the energy-costly and slow de novo synthesis and assembly of the entire PSI complex [67]. The particular susceptibility of PSI Fe-S clusters (Fx, FA, FB) stems from the nature of the attacking ROS and the limitations of the chloroplast antioxidant repertoire. Under over-reduction conditions, PSI primarily generates O_2_•^−^ at the Fe-S clusters via the Mehler reaction. While SOD rapidly converts O_2_•^−^ to H_2_O_2_, this reaction occurs in the stromal phase. The Fe-S clusters are deeply embedded within the PSI complex, creating a microenvironment where locally produced O_2_•^−^ can directly attack the clusters before being scavenged by stromal SOD. Furthermore, the highly oxidizing •OH, which can be formed from H_2_O_2_ via Fenton reactions, is virtually impossible for any enzymatic system to scavenge at diffusion-limited rates and is catastrophically damaging to Fe-S centers. Therefore, while the ascorbate–glutathione cycle effectively manages bulk stromal H_2_O_2_, it cannot provide site-specific, instantaneous protection to the PSI reaction center against the initial burst of O_2_•^−^. This makes prevention of over-reduction through alternative electron sinks a more effective strategy than post-facto detoxification for PSI protection [68,69]. This vulnerability is particularly acute under fluctuating light conditions. Here, the rapid transitions from low to high light can create sudden acceptor-side limitation at PSI. If the protective PGR5/PGRL1-mediated CEF is insufficient or slow to induce photosynthetic control, PSI is exposed to damaging electron backflow, leading to irreversible photoinhibition [58,70].

This striking disparity between the facile repair of PSII and the vulnerability of PSI to permanent damage has profound implications for the plant’s overall stress response strategy. It explains why investment in preventative mechanisms, particularly those that protect PSI, such as CEF and the WWC, is evolutionarily favored over reliance on post-damage repair [15,58]. Furthermore, it reveals that the “repair dimension” is not merely a clean-up operation but a critical determinant of the threshold at which the system transitions from the reversible damage-acclimation cycle to the irreversible damage-cell death cascade described in Section 3.1. Understanding this threshold, and the differential vulnerability of the two photosystems, is essential for predicting plant performance under dynamic field conditions and for engineering crops with enhanced resilience [56,71].

### 4.3. Ecological and Evolutionary Context of Photo-Oxidative Resilience

The molecular mechanisms governing photo-oxidative stress are not uniform across the plant kingdom but are instead evolutionary products fine-tuned by ecological niche and life history strategy. This natural variation reveals fundamental trade-offs and provides a crucial genetic reservoir for breeding resilient crops.

A primary evolutionary trade-off exists between maximizing growth under limiting light and minimizing damage under excess light. Species adapted to high-light environments, such as sun-acclimated plants, typically exhibit constitutively elevated capacities for NPQ, antioxidant pools, and CEF [47,49,60,64]. In contrast, shade-adapted species prioritize efficient light capture but possess minimal photoprotective reserves, leaving them highly vulnerable to sudden high-light exposure [72]. This divergence in strategy underscores the high metabolic cost of photoprotection, which creates a pervasive growth–defense trade-off. Investment in antioxidants, photoprotective pigments like zeaxanthin, and protein repair cycles (e.g., D1 turnover) diverts resources from growth and reproduction. Consequently, genetic lines with enhanced stress tolerance, such as *Arabidopsis* mutants with elevated ^1^O_2_ signaling or antioxidant capacity, often exhibit reduced growth under non-stressful conditions [23].

Convergent evolutionary adaptations to minimize photo-oxidative stress are exemplified by C4 and Crassulacean Acid Metabolism (CAM) photosynthesis. Both pathways function as preemptive solutions by concentrating CO_2_ around Rubisco, thereby dramatically suppressing photorespiration (a major source of H_2_O_2_) and minimizing conditions that lead to PETC over-reduction [73]. C4 plants, with their specialized Kranz anatomy, can maintain high photosynthetic rates with partially closed stomata under drought and heat, conferring superior resilience to combinatorial stresses that induce photo-oxidation. CAM plants achieve similar benefits by temporally separating CO_2_ fixation (at night) from light capture (during the day), thereby avoiding transpirational water loss and minimizing photoinhibitory risk.

Understanding this ecological and evolutionary context is directly applicable to crop improvement. The vast natural variation between stress-tolerant wild relatives and high-yielding cultivars represents a genetic resource for identifying valuable alleles. Targeted screening of germplasm for traits such as rapid NPQ relaxation, robust CEF activity, and stable PSI function under fluctuating light can uncover these alleles [56,74]. The challenge for modern breeding lies in leveraging these evolved natural solutions informed by the ecological context from which they arose to engineer climate-resilient crops that mitigate the yield penalty traditionally associated with enhanced photoprotection.

This ecological perspective reveals that the trade-off between growth and photoprotection is not an engineering problem to be eliminated, but a fundamental biological constraint that must be managed. Section 4.4 examines how this understanding informs current efforts to engineer crop resilience.

### 4.4. Harnessing Photoprotection for Crop Improvement

The unified model of photo-oxidative stress developed in preceding sections, with its interconnected signaling networks, self-amplifying damage cycles, and multilayered defenses, has direct implications for engineering climate-resilient crops. Rather than asking “which single gene should we overexpress?”, this systems perspective forces us to ask “how can we optimize the behavior of the entire network?” Emerging strategies reflect this shift in thinking.

The fundamental understanding of photoprotective mechanisms has opened a promising frontier for engineering climate-resilient crops. Moving beyond proof-of-concept in model plants, the focus is now on translating these mechanisms into agronomically viable traits that enhance yield under real-world field conditions. Several strategies beyond the landmark engineering of accelerated NPQ relaxation have shown significant promise. These include the targeted overexpression of chloroplast antioxidant enzymes, such as SOD and APX, to mitigate photo-oxidative damage across multiple stresses [75]. Rewiring photorespiration through synthetic glycolate metabolic pathways, which bypass the native H_2_O_2_-producing route, has successfully reduced energy losses and increased biomass in crops like tobacco and rice [76]. Furthermore, enhancing alternative electron sinks by manipulating components like PGR5 to boost CEF has proven effective in protecting PSI and improving growth under fluctuating light [15].

However, translating these successes from controlled environments to robust performance in major crops under field conditions remains a significant challenge. A critical evaluation reveals that the frequent failure of CRISPR-edited or transgenic crops with enhanced antioxidant capacity in field trials, compared to their promising greenhouse performance, stems from several interconnected factors rooted in environmental complexity and physiological trade-offs:

Stress complexity: The unified model reveals that the stress response system evolved to recognize specific ROS signatures [11,20]. Greenhouse trials using single, constant stresses activate only a subset of this recognition network, whereas field conditions engage the full system, sometimes in conflicting ways [71,77].

Pleiotropy: Because the redox network is highly interconnected [62,63], perturbing a single node (e.g., overexpressing APX) alters the entire system’s behavior. In the greenhouse, this may appear beneficial; in the field, unforeseen connections to development [77] or hormone signaling [39] can emerge.

Resource cost: The growth–defense trade-off documented in Section 4.3 [23,56] is not a design flaw but an evolved optimization. Constitutive defense expression consumes resources that, in a competitive field environment, directly reduce yield [71,78].

Temporal misregulation: The tiered defense system described in Section 4.1 operates on specific timescales. Constitutive expression disrupts this temporal coordination, potentially activating costly defenses when they are not needed or failing to activate them rapidly enough when they are [56].

Therefore, future engineering strategies must move beyond single-gene approaches and consider the resilient performance of the entire redox network under multifactorial stress. This necessitates field-based phenotyping and selection from the earliest stages of biotech pipeline development, using metrics that integrate yield with resilience, to identify strategies that are robust in the face of environmental complexity [71,77,78].

## 5. Future Research Directions

Advances in understanding the molecular mechanisms of photo-oxidative stress have opened new frontiers for predictive modeling and targeted engineering of plant resilience under climate change. Future research will likely converge on several interdisciplinary approaches that bridge fundamental discovery with agricultural application. These future directions are not equally urgent. We propose a three-phase roadmap informed by the unified model:

Phase 1: Mapping Network Topology. Single-cell omics [79] and spatially resolved biosensors [3,16] are urgently needed to map which cell types express which signaling components, revealing the spatial architecture of the stress response network.

Phase 2: Quantifying Network Dynamics. Temporal multi-omics [80] and real-time biosensor imaging will capture how the network behaves over time during stress onset and recovery, distinguishing causal drivers from downstream responses.

Phase 3: Predictive Network Engineering. With network topology and dynamics characterized, CRISPR-based editing [76] and synthetic biology can be deployed to optimize network behavior rather than single nodes, informed by GWAS-identified natural variants [74,81] that have already solved the growth–defense trade-off.

In conclusion, the unified model of photo-oxidative stress presented here, integrating ROS signaling, damage propagation, and multilayered defense, provides both a conceptual framework and an actionable roadmap. By treating stress resilience as an emergent property of a complex network rather than a collection of individual traits, we can move beyond the failures of single-gene engineering toward truly robust, climate-resilient crop varieties. The tools to map, model, and manipulate this network are now at hand; the challenge is to deploy them with the systems-level perspective they demand.

## Figures and Tables

**Table 1:** Key enzymatic and non-enzymatic components of the plant antioxidant network, their subcellular localization, and primary ROS targets.

Antioxidant	Class	Major Subcellular Localization	Primary ROS Target(s)	Key Function/Notes
Superoxide Dismutase (SOD)	Enzymatic	Cu/Zn-SOD: Chloroplast stroma, Cytosol, Peroxisome, Apoplast. Fe-SOD: Chloroplast stroma. Mn-SOD: Mitochondrial matrix.	O_2_•^−^ (Superoxide anions)	First line of defense; dismutates O_2_•^−^ to H_2_O_2_ and O_2_. Isoform localization dictates site-specific protection.
Ascorbate Peroxidase (APX)	Enzymatic	tAPX: Thylakoid membrane. sAPX: Chloroplast stroma. cAPX: Cytosol. pAPX: Peroxisome.	H_2_O_2_ (Hydrogen Peroxide)	Central to AsA-GSH cycle; reduces H_2_O_2_ to water using ascorbate. Different isoforms manage compartment-specific H_2_O_2_ bursts.
Catalase (CAT)	Enzymatic	Predominantly peroxisomes (glyoxysomes).	H_2_O_2_	High-capacity H_2_O_2_ removal without reductant consumption; crucial for photorespiratory H_2_O_2_ detoxification.
Glutathione Peroxidase (GPX)	Enzymatic	Chloroplast, Cytosol, Mitochondria, Endoplasmic Reticulum.	H_2_O_2_, Organic Hydroperoxides (e.g., lipid peroxides)	Uses glutathione (GSH) to reduce peroxides; links H_2_O_2_ metabolism to GSH redox state.
Peroxiredoxin (PRX)	Enzymatic	2-Cys PRX, PRX Q: Chloroplast. Others: Cytosol, Mitochondria.	H_2_O_2_, Organic Hydroperoxides, Peroxynitrite	Thiol-based peroxidases; involved in redox signaling and chaperone function under high stress.
Ascorbate (AsA)	Non-enzymatic	Chloroplast (highest concentration), Cytosol, Apoplast, Mitochondria.	•OH (Hydroxyl radical), ^1^O_2_ (Singlet oxygen), H_2_O_2_ (via APX)	Major soluble antioxidant; direct scavenger and essential co-factor for APX. Regenerated via the AsA-GSH cycle.
Glutathione (GSH)	Non-enzymatic	Chloroplast, Cytosol, Mitochondria, Nucleus.	•OH, ^1^O_2_, H_2_O_2_ (via GPX/AsA-GSH cycle)	Tripeptide thiol; maintains cellular redox homeostasis, regenerates ascorbate, detoxifies xenobiotics via GSTs.
α-Tocopherol (Vitamin E)	Non-enzymatic	Thylakoid membranes (lipid phase).	^1^O_2_, Lipid peroxyl radicals (LOO•)	Lipid-soluble antioxidant; protects PUFAs in membranes from lipid peroxidation chain reactions.
Carotenoids (β-Carotene, Xantho-phylls)	Non-enzymatic	Thylakoid membranes (bound to LHCs).	^1^O_2_, ^3^Chl* (Triplet chlorophyll)	Quench excess excitation energy and scavenge ^1^O_2_ directly; xanthophylls (zeaxanthin) central to NPQ.
Flavonoids	Non-enzymatic	Vacuole, Cell wall, Cytosol, Nucleus.	O_2_•^−^, •OH, H_2_O_2_ (via peroxidase action)	Diverse phenolic compounds; scavenge ROS, chelate pro-oxidant metals, and contribute to UV-B protection.

Abbreviations: LHCs, Light-Harvesting Complexes; NPQ, Non-Photochemical Quenching; PUFAs, Polyunsaturated Fatty Acids.

## Data Availability

No new data were created or analyzed in this study. Data sharing is not applicable to this article.

## Abbreviations

The following abbreviations are used in this manuscript:

•OH	Hydroxyl radicals
^1^O_2_	Singlet oxygen
^3^Chl	Triplet state of chlorophyll
β-CC	β-cyclocitral
ABA	Abscisic acid
APX	Ascorbate peroxidase
AsA-GSH	Ascorbate–glutathione
CAM	Crassulacean Acid Metabolism
CEF	Cyclic electron flow
EX1	Nuclear-encoded chloroplast proteins EXECUTER1
EX2	Nuclear-encoded chloroplast proteins EXECUTER2
GWAS	Genome-wide association studies
GST	Glutathione S-transferase
H_2_O_2_	Hydrogen peroxide
HSPs	Heat shock proteins
JA	Jasmonic acid
MDA	Malondialdehyde
MEcPP	Methylerythritol cyclodiphosphate
MSRs	Methionine sulfoxide reductases
NPQ	Non-photochemical quenching
NTRC	NADPH-thioredoxin reductase C
O_2_•^−^	Superoxide anions
PAP	3′-phosphoadenosine 5′-phosphate
PETC	Photosynthetic electron transport chain
PRXs	Peroxiredoxins
PSI	Photosystem I
PSII	Photosystem II
PSBS	PSII Subunit S
PUFAs	Polyunsaturated fatty acids
RBOH	Respiratory burst oxidase homolog
RES	Reactive electrophilic species
ROS	Reactive oxygen species
SA	Salicylic acid
SAA	Systemic acquired acclimation
SAR	Systemic acquired resistance
Trx	Thioredoxin
UPS	Ubiquitin–proteasome system
WWC	Water–water cycle
